# Fukutin regulates tau phosphorylation and synaptic function: Novel properties of fukutin in neurons

**DOI:** 10.1111/neup.12797

**Published:** 2022-01-13

**Authors:** Ryota Tsukui, Tomoko Yamamoto, Yukinori Okamura, Yoichiro Kato, Noriyuki Shibata

**Affiliations:** ^1^ Graduate School of Medicine Tokyo Women's Medical University Tokyo Japan; ^2^ Division of Human Pathology & Pathological Neuroscience, Department of Pathology Tokyo Women's Medical University Tokyo Japan; ^3^ Department of Surgical Pathology Tokyo Women's Medical University Tokyo Japan

**Keywords:** fukutin, GABA, GSK‐3β, synaptophysin, tau

## Abstract

Fukutin, a product of the causative gene of Fukuyama congenital muscular dystrophy (FCMD), is known to be responsible for basement membrane formation. Patients with FCMD exhibit not only muscular dystrophy but also central nervous system abnormalities, including polymicrogyria and neurofibrillary tangles (NFTs) in the cerebral cortex. The formation of NFTs cannot be explained by basement membrane disorganization. To determine the involvement of fukutin in the NFT formation, we performed molecular pathological investigations using autopsied human brains and cultured neurons of a cell line (SH‐SY5Y). In human brains, NFTs, identified with an antibody against phosphorylated tau (p‐tau), were observed in FCMD patients but not age‐matched control subjects and were localized in cortical neurons lacking somatic immunoreactivity for glutamic acid decarboxylase (GAD), a marker of inhibitory neurons. In FCMD brains, NFTs were mainly distributed in lesions of polymicrogyria. Immunofluorescence staining revealed the colocalization of immunoreactivities for p‐tau and phosphorylated glycogen synthase kinase‐3β (GSK‐3β), a potential tau kinase, in the somatic cytoplasm of SH‐SY5Y cells; both the immunoreactivities were increased by *fukutin* knockdown and reduced by *fukutin* overexpression. Western blot analysis using SH‐SY5Y cells revealed consistent results. Enzyme‐linked immunosorbent assay (ELISA) confirmed the binding affinity of fukutin to tau and GSK‐3β in SH‐SY5Y cells. In the human brains, the density of GAD‐immunoreactive neurons in the frontal cortex was significantly higher in the FCMD group than in the control group. GAD immunoreactivity on Western blots of SH‐SY5Y cells was significantly increased by *fukutin* knockdown. On immunofluorescence staining, immunoreactivities for fukutin and GAD were colocalized in the somatic cytoplasm of the human brains and SH‐SY5Y cells, whereas those for fukutin and synaptophysin were colocalized in the neuropil of the human brains and the cytoplasm of SH‐SY5Y cells. ELISA confirmed the binding affinity of fukutin to GAD and synaptophysin in SH‐SY5Y cells. The present results provide *in vivo* and *in vitro* evidence for novel properties of fukutin as follows: (i) there is an inverse relationship between fukutin expression and GSK‐3β/tau phosphorylation in neurons; (ii) fukutin binds to GSK‐3β and tau; (iii) tau phosphorylation occurs in non‐GAD‐immunoreactive neurons in FCMD brains; (iv) neuronal GAD expression is upregulated in the absence of fukutin; and (v) fukutin binds to GAD and synaptophysin in presynaptic vesicles of neurons.

## INTRODUCTION

Fukuyama congenital muscular dystrophy (FCMD), the second most common muscular dystrophy in Japan, is characterized by congenital muscular dystrophy associated with congenital malformations of the central nervous system (CNS) and the eye.^1^, ^2^, ^3^ *Fukutin* is a gene responsible for FCMD.^4^ The gene product protein fukutin participates in the glycosylation of α‐dystroglycan (α‐DG) by transferring ribitol‐5‐phosphate to the sugar chain.^5^ α‐DG is involved in the formation of the basement membrane, and the glycosylated domain acts as a receptor for extracellular matrix proteins.^6^, ^7^ Therefore, the fragility of the basement membrane, resulting from reduced fukutin expression, causes muscular dystrophy.^8^ The representative malformation of the CNS associated with FCMD is polymicrogyria of the cerebral and cerebellar cortices,^9^, ^10^ where astrocytes deeply participate in the lesion formation.^11^, ^12^ In the CNS, the glia limitans is composed of the foot processes of astrocytes and the covering basement membrane,^13^ associated with the coexistence of the dystrophin‐glycoprotein complex (DGC).^14^ Altered functions of fukutin in astrocytes lead to basement membrane disorganization and fragility that gives rise to the disruption of the glia limitans. During the fetal period, neurons and glia overmigrate into subarachnoid space via the disrupted glia limitans to form so‐called “gliomesenchymal tissue”, and the secondarily occurring fuse between the adjacent cortices results in polymicrogyria.^15^

It is known that fukutin is also expressed in neurons.^12^, ^16^, ^17^ Considering higher expression levels of fukutin in immature neurons as compared to mature neurons, it is predicted that fukutin is essential for the migration of immature neurons.^17^ In contrast, it is suggested that fukutin participates in the synaptic function of mature neurons;^17^ however, the mechanism has not been fully clarified. In FCMD, neurofibrillary tangles (NFTs), intracellular aggregates of phosphorylated tau (p‐tau), are observed in “mature neurons” of elderly patients' brains, particularly around 30 years old,^18^, ^19^ whereas senile plaques, as amyloid‐β (Aβ) deposits, are absent.^18^, ^19^ In FCMD, NFTs are distributed in the cerebral neocortex, limbic system, and brainstem.^18^ In Alzheimer's disease (AD), senile plaques appear in the limbic system and spread across the whole cerebral region,^21^ followed by the formation of NFTs.^21^ Thus, the formation processes of NFTs in FCMD brain differ from those in AD brain and remain to be determined. To address this issue, we focused on the relationship between fukutin expression status and tau phosphorylation status. We also paid attention to implications for fukutin in synaptic function, as suggested in a previous study.^17^ The DGC exists in the postsynaptic terminal and is related to the postsynaptic function.^22^ However, only a few studies have been conducted on presynaptic DGC.^23^, ^24^, ^25^ Therefore, we investigated the involvement of fukutin in synaptic function. This is the first report to demonstrate *in vivo* and *in vitro* evidence of novel properties of fukutin in neurons, using immunohistochemistry, immunocytochemistry, Western blotting, and enzyme‐linked, immunosorbent assay (ELISA).

## MATERIALS AND METHODS

### Human subjects

This investigation was carried out on archival, formalin‐ or UFIX (Sakura, Tokyo)‐fixed, paraffin‐embedded tissues of brains obtained at autopsy from three FCMD patients (two male and one female, aged 13–27 years) and three age‐matched control subjects (one male and two female). Their clinical features are summarized in Table 1.

**Table 1:** Clinical features of cases examined

Case	Disease	Age at death	Sex	Postmortem time	Brain weight (g)
1	FCMD	13 years	M	3 h 30 min	1336
2	FCMD	17 years	F	5 h 29 min	1340
3	FCMD	27 years	M	1 h 32 min	1311
4	SLE	17 years	F	14 h 50 min	1420
5	VAHS	29 years	M	1 h 37 min	1418
6	MS	31 years	F	7 h 4 min	1415

F, female; FCMD, Fukuyama congenital muscular dystrophy; h, hour; M, male; min, minute; MS, Marfan syndrome; SLE, systemic lupus erythematosus; VAHS, virus‐associated hemophagocytotic syndrome.

### Immunohistochemistry

Immunohistochemistry was performed with primary antibodies against fukutin (rabbit polyclonal, Cat. No. N3C3–2; GeneTex, Irvine, CA, USA; 1:500), p‐tau (mouse monoclonal, clone AT8; Fujirebio Europe NV, Gent, Belgium; 1:5000), GAD (rabbit monoclonal, clone EPR19366; Abcam, Cambridge, UK; 1:500), tau (mouse monoclonal, clone 2B11; Immuno‐Biological Laboratories, Gunma, Japan; 1:20), GAD‐65/67 (mouse monoclonal, clone C‐9; Santa Cruz Biotechnology, Santa Cruz, CA, USA; 1:10), and synaptophysin (mouse monoclonal, clone SY38; Dako, Glostrup, Denmark; 1:100).

Multiple 6‐μm‐thick sections of the control and FCMD brains were deparaffinized and rehydrated. Unmasking of all the examined antigens was conducted by microwaving the sections for 40 min in 1 mM ethylendiaminetetraacetic acid (EDTA)/tris(hydroxymethyl)aminomethane (Tris) buffer, pH 9.0. Subsequently, sections were quenched with 3% H_2_O_2_ for 10 min at room temperature (RT) to inhibit endogenous peroxidase activity, rinsed in phosphate‐buffered saline (PBS), pH 7.6, pretreated with 5% skim milk/PBS solution for 30 min at RT to block nonspecific antibody binding, and incubated overnight at 4°C with the primary antibodies mentioned above. Immunoreaction product deposits were visualized by the polymer‐immunocomplex method using the respective Histofine Simple Stain Polymer kits (Nichirei, Tokyo, Japan). 3,3′‐Diaminobenzidine tetrahydrochloride (DAB) (Dojindo, Kumamoto, Japan) was the chromogen, and hematoxylin, the counterstain. Sections from which the primary antibodies were omitted served as negative reaction controls.

Light microscopic double‐labeled immunohistochemical staining was conducted to compare tissue localizations of p‐tau and GAD. In brief, sections were deparaffinized, rehydrated, quenched with the H_2_O_2_ solution, pretreated with the skim milk solution, and incubated overnight at 4°C with the anti‐p‐tau antibody. Antibody binding for p‐tau was detected by the polymer‐immunocomplex method, as mentioned above, with DAB (brown) as the chromogen. The sections were subsequently processed by microwaving for 40 min in the Tris‐EDTA solution for the purpose of eluting deposited antigen–antibody complexes as well as unmasking GAD antigen. Sections were then rinsed in PBS and incubated overnight at 4°C with the anti‐GAD antibody. Antibody binding for GAD was detected using the polymer‐immunocomplex method, as mentioned above, with DAB/NiCl_2_ (indigo) as the chromogen. Double‐stained sections were microphotographed by light microscopy. Omission of the primary antibodies on sections gave negative reaction controls.

For immunofluorescence staining, antibody binding was visualized using the respective secondary antibodies: Alexa Fluor 488‐conjugated donkey anti‐mouse IgG H + L (Cat. No. A‐21202; Thermo Fisher Scientific, Waltham, MA, USA; 1:500) and Alexa Fluor 555‐conjugated donkey anti‐rabbit IgG H + L (Cat. No. A‐31572; Thermo Fisher Scientific; 1:500). Cell nuclei were counterstained with 4′,6‐diamidino‐2‐phenylindole (DAPI) (Vector Laboratories, Burlingame, CA, USA). Slides with omission of the primary antibodies gave negative reaction controls. Immunostained slides were observed using a fluorescence microscope (Nikon ECLIPSE TS100; Nikon, Tokyo, Japan).

### Cell culture

The human neuroblastoma cell line (SH‐SY5Y) was used in this investigation. SH‐SY5Y cells were grown in Dulbecco's modified Eagle's medium (DMEM) (Cat. No. 11995065; Thermo Fisher Scientific) supplemented with 10% fetal bovine serum (FBS) (Thermo Fisher Scientific) and 1% penicillin–streptomycin (Thermo Fisher Scientific). Cells were incubated in a Falcon 25‐cm^2^ Rectangular Canted Neck Cell Culture Flask with Vented Cap (Cat. No. 353108; Corning, Corning, NY, USA) or a Falcon four‐well Culture Slide (Cat. No. 354114; Corning) and maintained at 37°C in a humidified incubator under 5% CO_2_ atmosphere. Cell counting was verified by the trypan blue dye exclusion method using a LUNA‐FL automated cell counter (Logos Biosystems, Gyeonggi‐do, South Korea) and dedicated using LUNA Cell Counting Slides (Logos Biosystems). SH‐SY5Y cells were divided into different groups with or without several treatments as mentioned later.

### Knockdown of *fukutin*

Stealth short‐hairpin RNA (siRNA) duplexes against *fukutin* mRNA were designed and synthesized by Thermo Fisher Scientific. The target sense for *fukutin* mRNA was 5′‐UUUUGAAGGGAACAAAUUUCCUGUC‐3′ (F697). As a scramble, Silencer Negative Control No. 1 siRNA (Cat. No. AM4611; Thermo Fisher Scientific) was used, and omission of siRNA gave a negative reaction control (vehicle). SY‐SH5Y cells were plated at a density of approximately 5 × 10^5^ cells in a 25‐cm^2^ flask or 1 × 10^4^ cells in a 1.7‐cm^2^ chamber slide one day before siRNA transfection. At a final concentration of 40 nM, siRNA was transfected into the cells using a Lipofectamine MAX (Thermo Fisher of Scientific) and Opti‐MEM (Thermo Fisher Scientific) according to the manufacturer's instructions. Four days after transfection, cells on slides were fixed in 100% methanol for fluorescence immunocytochemistry, and cells on flasks were harvested for Western blotting and sandwich ELISA.

### Neural differentiation of SH‐SY5Y cells

In a preliminary study, immunoreactivities for p‐tau and glycogen synthase kinase‐3β (GSK‐3β) phosphorylated at codon 216 tyrosine residue (p‐GSK‐3β) were undetectable in SH‐SY5Y cells. Based on this fact, we considered that neural differentiation was necessary to detect both these proteins. SH‐SY5 cells were plated at a density of approximately 2.5 × 10^5^ cells in a 25‐cm^2^ flask. Retinoic acid (all‐trans‐RA; Merck KGaA Sigma‐Aldrich, St. Louis, MO, USA) was dissolved in dimethyl sulfoxide. One day after plating, cells were incubated with the maintenance medium containing the retinoic acid solution at a final concentration of 10 μM for five days as described previously.^26^ Cells were then washed with PBS, harvested using 0.25% trypsin (Thermo Fisher Scientific), and seeded on a slide for fluorescence immunocytochemistry.

### Overexpression of *fukutin*

For overexpression of *fukutin*, the open reading frame of *fukutin* cDNA was cloned into pcDNA 3.1^+^‐DYK tag (OHu23291) by GenScript (Piscataway, NJ, USA). SH‐SY5 cells were plated at a density of approximately 2.5 × 10^4^ cells in a 1.7‐cm^2^ chamber slide one day before plasmid transfection. The plasmids, harboring a large amount of *fukutin* cDNA, were transfected into SH‐SY5Y cells using a Lipofectamine 3000 (Thermo Fisher Scientific) and an Opti‐MEM (Thermo Fisher Scientific). Green‐fluorescence protein (GFP)‐labeled pcDNA3.1 was used as a transgene control. Twelve hours after plasmid transfection, the medium was changed to the maintenance medium without plasmids. Successful transfection was confirmed by detection of luminescence of GFP. Two days after transfection, cells were used for immunofluorescence staining.

### Immunocytochemistry

SH‐SY5Y cells on a slide were fixed in 100% methanol for 15 min at RT, pretreated with 0.2% Triton X‐100 in PBS for 15 min, treated with 5% skim milk in PBS for 30 min at RT, and subsequently incubated overnight at 4°C with the primary antibodies mentioned above against fukutin (Cat. No. N3C3‐2; 1:500), p‐tau (clone AT8; 1:20–50), tau (clone 2B11; 1:20‐100), GSK‐3β (mouse monoclonal, clone 3D10; Cell Signaling Technology, Danvers, MA, USA; 1:100), p‐GSK‐3β (rabbit polyclonal, Cat.No.44‐604G; 1:100), GAD‐65/67 (clone C‐9; Santa Cruz Biotechnology; 1:10), and synaptophysin (clone SY38; 1:10). Cells were incubated overnight at 4°C with the primary antibodies, followed by incubation for 1 h at RT with the respective secondary antibodies: Alexa Fluor 488‐conjugated donkey anti‐mouse IgG (H + L) (Cat. No. A‐21202; 1:500) and Alexa Fluor 555‐conjugated donkey anti‐rabbit IgG (H + L) (Cat. No. A‐31572; 1:500). Cell nuclei were counterstained with DAPI. Slides with omission of the primary antibodies gave negative reaction controls. Immunostained slides were observed using the fluorescence microscope.

### Protein extraction

For Western blotting and sandwich ELISA, total protein extracts were obtained from SH‐SY5Y cells. In brief, cells were collected and suspended with ice‐cooled lysis buffer, consisting of 50 mM Tris–HCl, pH 7.4, 150 mM NaCl, 1 mM EDTA, 1% Triton X‐100, protease inhibitor cocktail (Complete Mini; Roche Diagnostics, Mannheim, Germany), and phosphatase inhibitor cocktail (PhosSTOP; Roche Diagnostics) for 30 min with occasional pipetting. The samples were centrifuged at 13 500 *g* for 30 min at 4°C, and the supernatant was used.

### Western blotting

Western blotting was performed using the primary antibodies against p‐tau (clone AT8; 1:50), tau (rabbit polyclonal, Cat. No. A0024; Dako; 1:1000), p‐GSK‐3β (Cat. No.44‐604G; 1:5000), GSK‐3β (clone 3D10; 1:1000), GAD‐65/67 (clone C‐9; 1:100), and glyceraldehyde‐3‐phosphate dehydrogenase (GAPDH) (rabbit monoclonal, clone 14C10; Cell Signaling Technology; 1:5000). The secondary antibodies were goat anti‐mouse IgG conjugated to horseradish peroxidase (HRP; Cat. No. sc‐2055; Santa Cruz Biotechnology; 1:5000) and donkey anti‐rabbit IgG conjugated to HRP (Cat. No. NA934‐1ML; Cytiva, Tokyo, Japan).

Protein samples (aliquot of 50 μg per lane) were electrophoresed on a 10% Mini‐PROTEAN TGX Gel (Bio‐Rad Laboratories, Hercules, CA, USA) and electrotransferred to a polyvinylidene difluoride (PVDF) membrane (Trans‐Blot Turbo 0.2 μm PVDF Membrane; Bio‐Rad). Blotted membranes were treated with a Can Get Signal/PVDF Blocking Reagent (TOYOBO, Tokyo, Japan) for 1 h, washed with 0.2% Tween‐20 in Tris‐buffered saline (TBS; 10× TBS; Cat. No. 1706435; Bio‐Rad) containing a Polyoxyethylene^20^ Sorbitan Monolaurate (FUJIFILM Wako Pure Chemical, Osaka, Japan), and incubated overnight at 4°C with the abovementioned primary antibodies diluted in a Can Get Signal Solution 1 (TOYOBO). Blots were then rinsed in 0.2% Tween‐20/TBS and incubated for 1 h at RT with the respective secondary antibodies diluted in Can Get Signal Solution 2 (TOYOBO). Immunoreactive signals were visualized by the chemiluminescence method using a Chemilucent Plus (Merck Millipore, Burlington, MA, USA) and photographed using a ChemiDoc XRS Plus system (Bio‐Rad). The optical density of each signal band was quantitatively measured using Image Lab software (Bio‐Rad).

### Sandwich ELISA

Microtiter plates were incubated for 1 h at 37°C with the anti‐fukutin antibody (Cat. No. N3C3‐2; 1:500) followed by washing with TBS. Subsequently, lysis buffer‐extracted total protein extracts from SH‐SY5Y cells were applied overnight at 4°C on a microplate at graded protein concentrations. After washing in TBS, microplates were treated for 1 h at RT with 5% skim milk in PBS, followed by washing with TBS. The plates were then incubated overnight at 4°C with a primary antibody against tau (clone 2B11; 1:50), GSK‐3β (clone 3D10; 1:500), GAD (clone C‐9; 1:100), or synaptophysin (clone SY38; 1:500). After washing with TBS, microplates were incubated with the secondary horse antibody HRP‐conjugated anti‐mouse IgG (H + L) (Vector Laboratories, Burlingame, CA, USA; 1:1000) for 1 h at RT. Each hybridized signal was detected using an Alkaline Phosphatase Yellow (qNPP) Liquid Substrate System (Sigma‐Aldrich). The plates were incubated for 24 h at RT, and titer was determined using a Thermo Scientific Multiskan GO Microplate (Thermo Fisher Scientific).

### Statistics

Values in individual groups were expressed as mean ± SEM. Comparison of the data among three groups was screened by one‐way analysis of variance (ANOVA), and two groups were compared by *post hoc* Bonferroni correction. Statistical significance was considered when the *P*‐value was less than 0.05.

## RESULTS

### Immunohistochemical observations for p‐tau in control and FCMD brains

**Fig 1:**
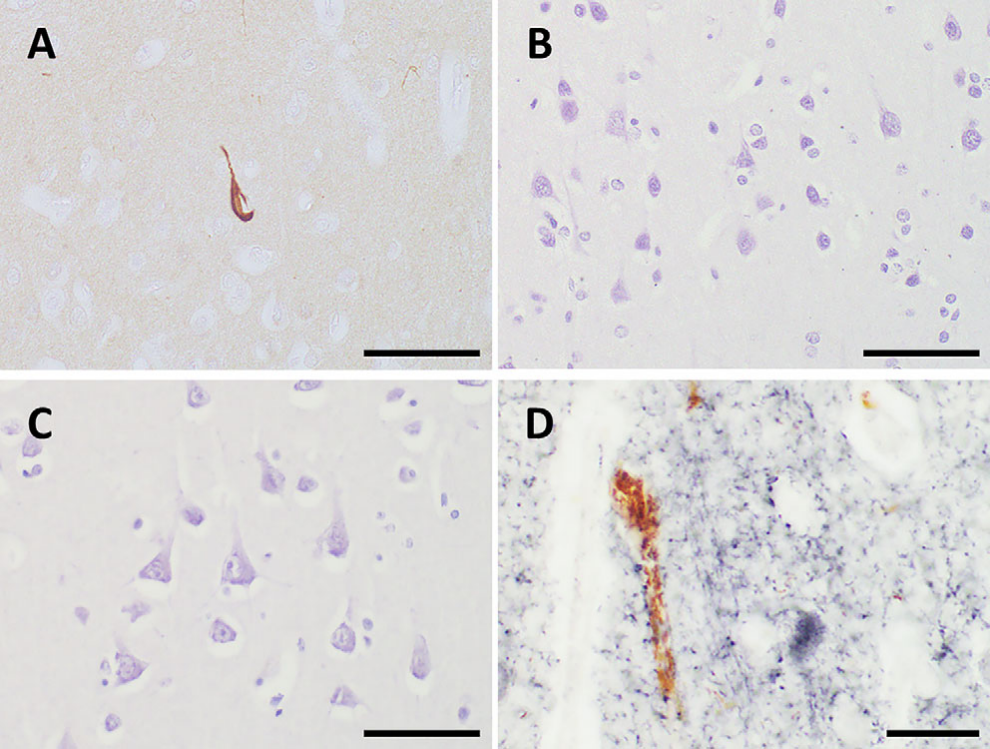
Representative findings of immunohistochemical localization for p‐tau in the cerebral cortex obtained at autopsy from a 27‐year‐old patient with FCMD (A, B, D) and a 29‐year‐old control subject (C). (A, B) Light microscopy identifies that cortical neurons immunoreactive for p‐tau are predominantly distributed in the frontal lobe (A) as compared to the occipital lobe (B). (C) By contrast, p‐tau immunoreactivity is only very weak or not observed in the control frontal cortex. (D) On double‐labeled staining, p‐tau immunoreactivity (brown by DAB) is exclusively localized in non‐GAD‐immunoreactive neurons in the FCMD brain, particularly in the frontal cortex. GAD‐immunoreactive neurons are positively stained with NiCl_2_/DAB (indigo). Scale bars: 50 μm (A–C), 20 μm (D).

On light microscopy, no immunoreaction product deposits were visible in negative reaction control sections (data not shown). Immunoreactivity for p‐tau in the cerebral cortex, including cortical dysplasia lesions, was distinct in the FCMD brains (Fig. 1A, B) but only very weak or not observed in the control brains (Fig. 1C). In the FCMD brains, p‐tau immunoreactivity was localized in the somatic cytoplasm of cortical neurons, and it was predominantly intense in the frontal lobe (Fig. 1A) as compared to the occipital lobe (Fig. 1B). The p‐tau‐immunoreactivity was localized in non‐GAD‐immunoreactive neurons (Fig. 1D) of the FCMD brains; both were exclusively distributed.

### Knockdown of *fukutin* induces phosphorylation of tau and GSK‐3β in SH‐SY5Y cells

**Fig 2:**
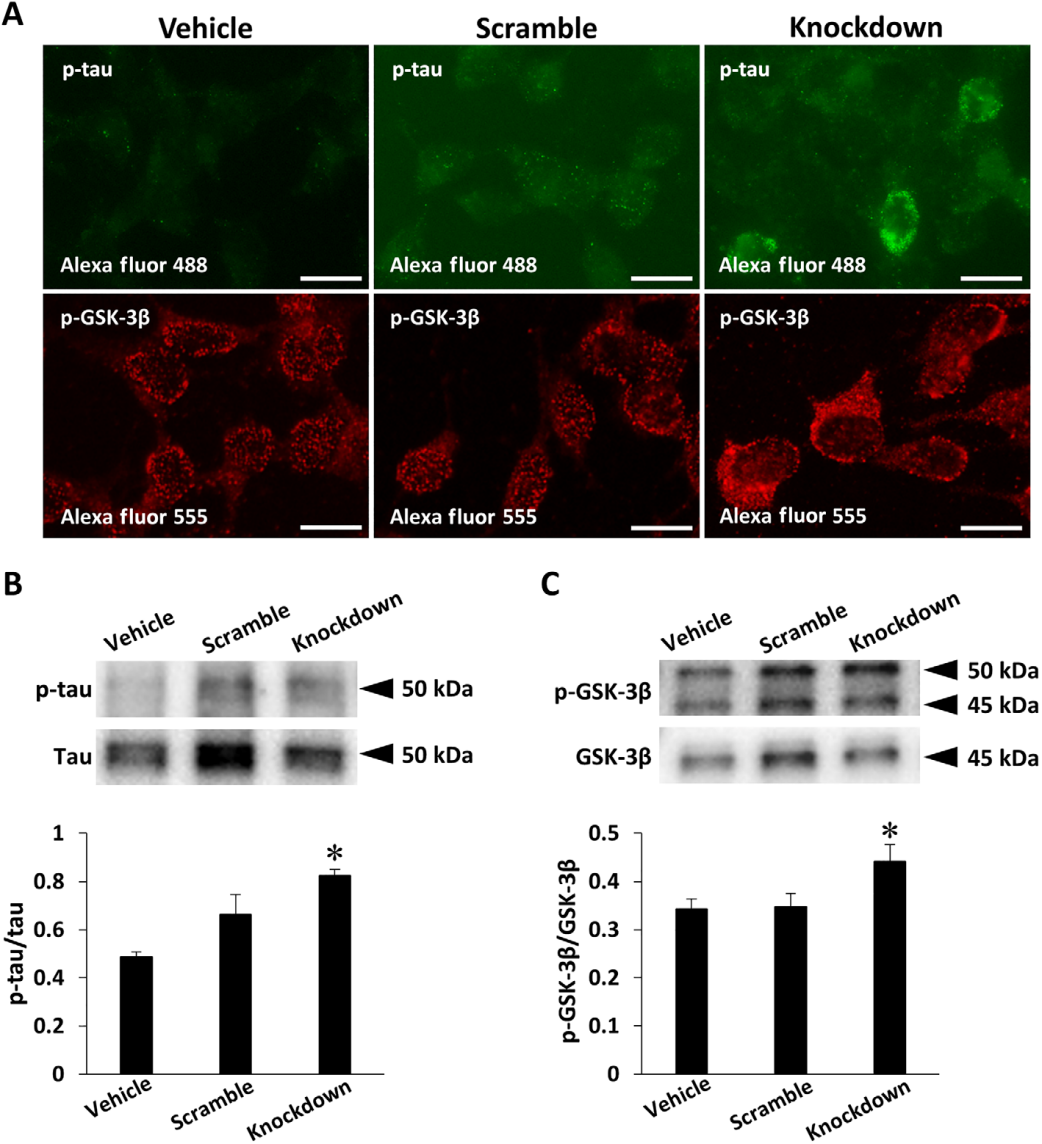
Results of immunocytochemistry (A) and Western blotting (B, C) for p‐tau and p‐GSK‐3β in SH‐SY5Ycells with or without *fukutin* knockdown. (A) On fluorescence microscopy, immunoreactivities for p‐tau (green) and p‐GSK‐3β (red) are distinct and localized in the somatic cytoplasm of cells of the knockdown group, in contrast to the vehicle and scramble groups showing cells only very weakly stained or not at all. (B, C) Immunoreactive signals for p‐tau (B) and p‐GSK‐3β (C) are detected in each lane at their predicted mobilities on blots. Both the p‐tau/tau optical density ratio and the p‐GSK‐3β/GSK‐3β optical density ratio are significantly increased in the knockdown group as compared to the vehicle and scramble groups. *P* < 0.05 on one‐way ANOVA; *P < 0.01 *vs* the vehicle group on *post hoc* Bonferroni correction. Scale bars: 20 μm (A).

Fluorescence immunocytochemistry was performed for morphological comparison of subcellular localizations of p‐tau and p‐GSK‐3β in SH‐SY5Y cells with or without *fukutin* knockdown. No immunoreactive signals were visible on negative reaction control slides (data not shown). Immunoreactive signals for p‐tau and p‐GSK‐3β were distinct and localized in the somatic cytoplasm of SH‐SY5Y cells of the *fukutin* knockdown group, and by contrast only very weak or not observed in the cells of the vehicle and scramble groups (Fig. 2A).

Western blot analysis was performed for quantitative comparison of p‐tau and p‐GSK‐3β in SH‐SY5Y cells with or without *fukutin* knockdown. No immunoreactive signals were visible on negative reaction control blots (data not shown). Immunoreactive signal bands for p‐tau (Fig. 2B) and p‐GSK‐3β (Fig. 2C) were detected at their respective predicted mobilities. Both the p‐tau/tau optical density ratio (Fig. 2B) and the p‐GSK‐3β/GSK‐3β optical density ratio (Fig. 2C) were significantly increased in the *fukutin* knockdown group as compared to the vehicle and scramble groups.

### Influence of *fukutin* overexpression on the phosphorylation status of tau and GSK‐3β in neural‐differentiated SH‐SY5Y cells

**Fig 3:**
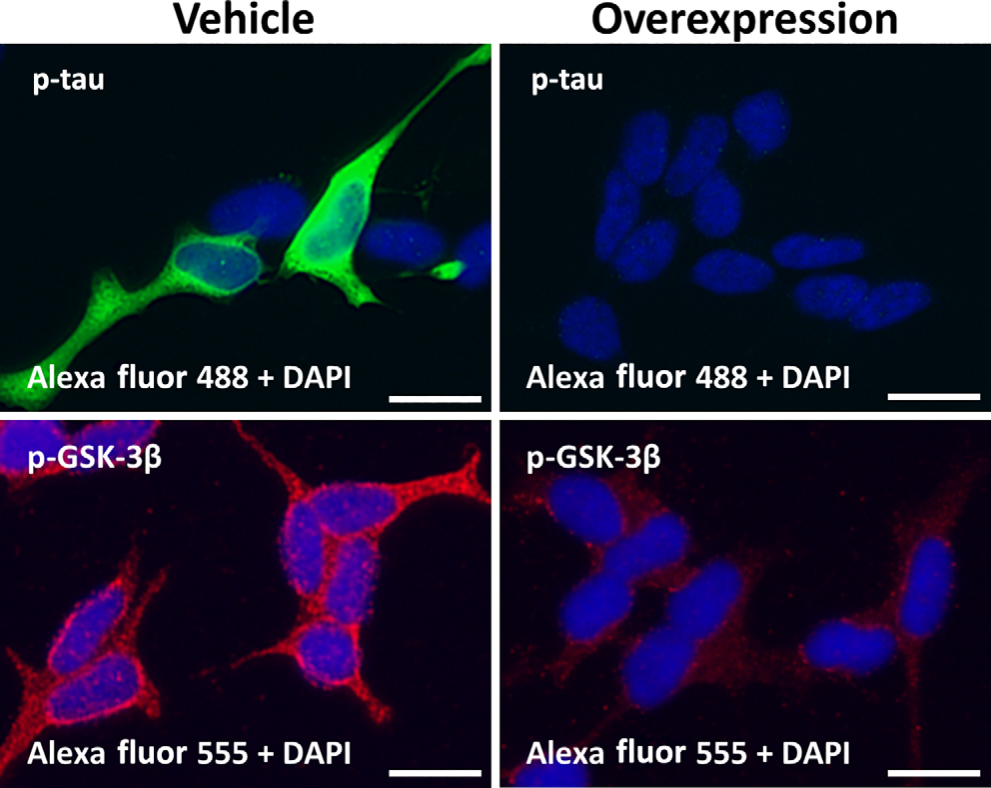
Immunocytochemical observations for p‐tau and p‐GSK‐3β in retinoic acid‐induced, neural‐differentiated SH‐SY5Y cells with or without *fukutin* overexpression. Fluorescence microscopy reveals that both p‐tau (green) and p‐GSK‐3β (red) immunoreactivities are localized in the somatic cytoplasm of vehicle cells but undetectable in overexpressed cells. Scale bars: 20 μm.

In our preliminary study, we predicted that *fukutin* overexpression would induce dephosphorylation of tau and GSK‐3β in SH‐SY5Y cells. However, both immunoreactivities for p‐tau and p‐GSK‐3β were only very weak in the cells. Therefore, we hypothesized that neural differentiation, induced by retinoic acid treatment, may be required for detecting p‐tau and p‐GSK‐3β. Consequently, both the immunoreactivities were proved to be distinct and localized in the somatic cytoplasm of retinoic acid‐induced, neural‐differentiated cells, whereas no significant immunoreactivities for them were detected in the neural‐differentiated cells with *fukutin* overexpression (Fig. 3).

### Fukutin is colocalized with tau and GSK‐3β in human brains and SH‐SY5Y cells

**Fig 4:**
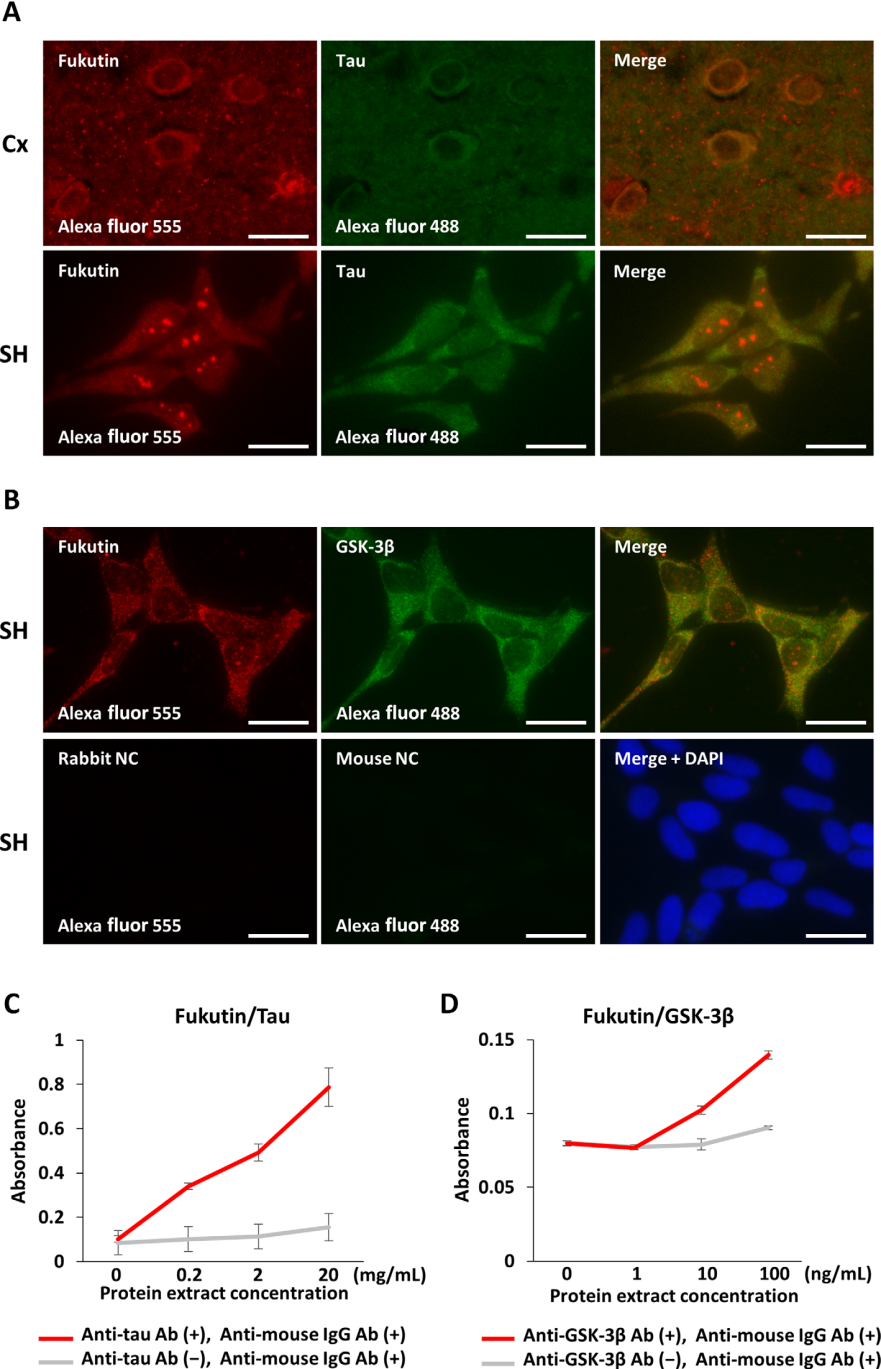
Representative findings of immunohistochemical and immunocytochemical localizations for fukutin, tau, or GSK‐3β in the cerebral cortex (Cx) from a 29‐year‐old control subject and SH‐SY5Y (SH) cells (A, B) as well as evidence of binding affinity of fukutin to tau or GSK‐3β in whole lysate of SH cells obtained by sandwich ELISA (C, D). (A, B) Fluorescence microscopy depicts the colocalization (yellow) of fukutin immunoreactivity (red) with tau immunoreactivity (green) in the somatic cytoplasm and neuropil of the Cx and in the cytoplasm of SH cells (A) as well as with GSK‐3β immunoreactivity (green) in the somatic cytoplasm of SH cells (B). No immunoreactive signals are visible on negative reaction control slides (B). (C, D) On sandwich ELISA, absorbances indicative of the formation of the fukutin/tau complex (C) and the fukutin/GSK‐3β complex (D) are significantly increased in a manner dependent on cell lysate concentrations of SH cells. *P* < 0.001 on one‐way ANOVA (C, D). Scale bars: 20 μm (A, B).

On double‐labeled immunofluorescence staining, we next compared immunohistochemical and immunocytochemical localizations of fukutin and tau or GSK‐3β in the control brains and SH‐SY5Y cells. Both fukutin and tau immunoreactivities were colocalized in the somatic cytoplasm and neuropil of the cerebral cortex and in the somatic cytoplasm of SH‐SY5Y cells (Fig. 4A). Similarly, both fukutin and GSK‐3β immunoreactivities were colocalized in the somatic cytoplasm of SH‐SY5Y cells (Fig. 4B). No immunoreactive signals were visible on negative reaction controls.

### Fukutin binds to tau and GSK‐3β in SH‐SY5Y cells

Given the colocalization data of fukutin and tau or GSK‐3β, we verified their binding affinity in SH‐SY5Y cells using a sandwich ELISA approach. Immunoreactive signals indicative of the formation of the fukutin/tau complex (Fig. 4C) and the fukutin/GSK‐3β complex (Fig. 4D) were significantly increased in a manner dependent on cell lysate concentrations. Both of these signals were canceled by omission of an antibody against tau or GSK‐3β.

### GAD‐immunoreactive neurons are predominantly distributed in the frontal lobe of FCMD brains

**Fig 5:**
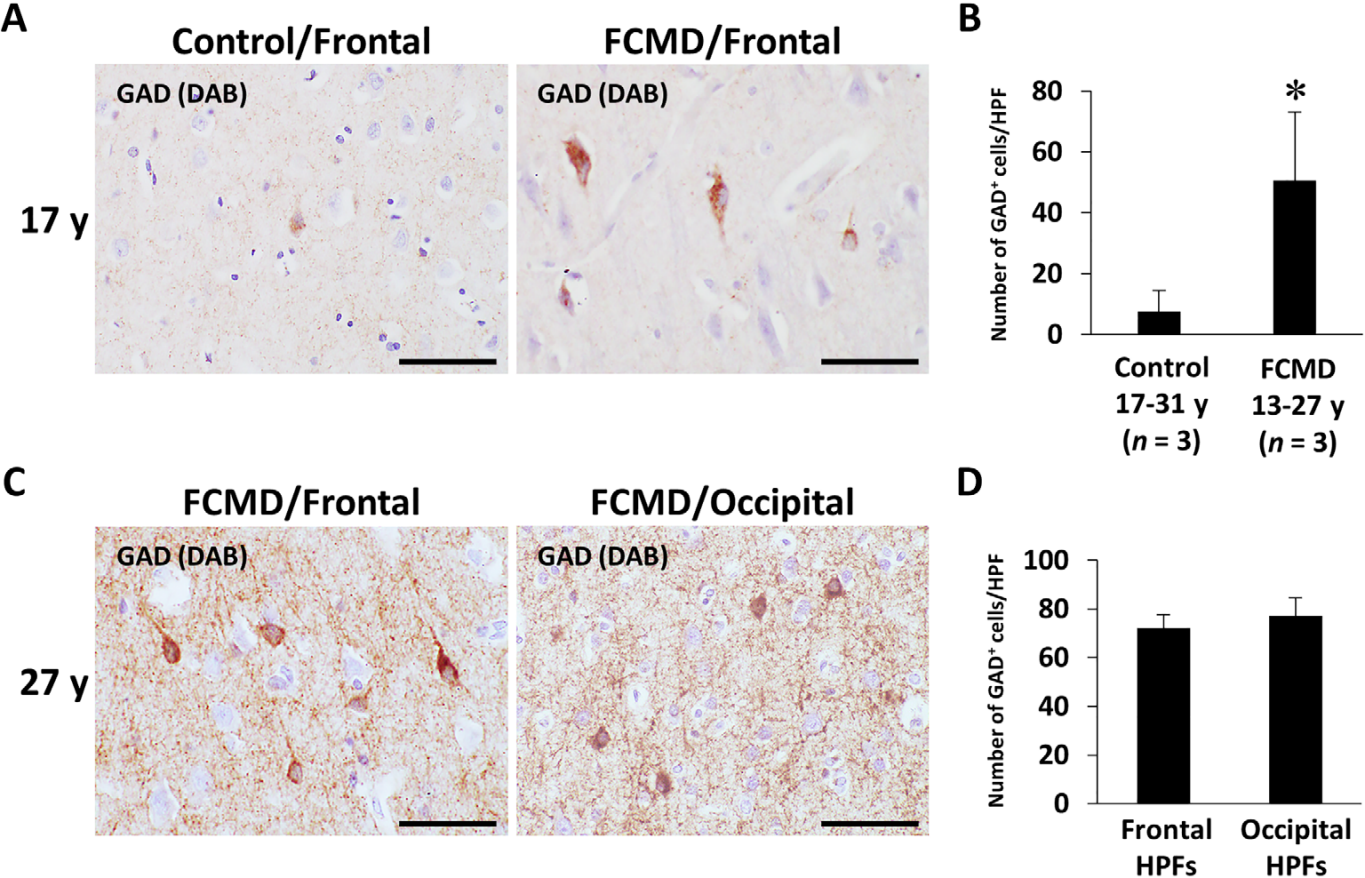
Representative findings of immunohistochemical localizations for GAD in the cerebral cortex from control and FCMD cases (A, C) as well as results of semiquantitative comparison of the density of GAD‐immunoreactive (GAD^+^) neurons between the different groups (B, D). (A) On light microscopy, the GAD^+^ neurons in the frontal lobe seem to be predominantly distributed in a 27‐year‐old FCMD patient as compared to a 29‐year‐old control subject. Staining for GAD is more intense in the FCMD brain than in the control brain. (B) The density of GAD^+^ neurons in the frontal cortex is significantly higher in the FCMD group as compared to the age‐matched control group. (C) In a 27‐year‐old FCMD patient, the distribution pattern of GAD^+^ neurons in the frontal lobe is similar to that in the occipital lobe. (D) There is no significant difference in the density of GAD^+^ neurons between the frontal and occipital lobe cortices of the same FCMD patient as in (C). HPF and HPFs indicate ten high‐power fields. y, years. *P < 0.05 *vs* the control group on unpaired Student's *t*‐test. Scale bars: 50 μm (A, C).

Light microscopy identified that GAD‐immunoreactive neurons in the cerebral cortex were predominantly distributed in the FCMD brains as compared to the age‐matched control brains (Fig. 5A). Semiquantitative analysis revealed that the density of GAD‐immunoreactive neurons in the frontal cortex was significantly higher in the FCMD group as compared to the control group (Fig. 5B). Immunohistochemically, in the FCMD brain, there was no significant difference in the distribution pattern of GAD‐immunoreactive neurons between the frontal and occipital cortices (Fig. 5C). Semiquantitatively, there was no significant difference in the density of GAD‐immunoreactive neurons between the frontal and occipital cortices of the FCMD brain (Fig. 5D). Staining for GAD in the neuropil was more intense in the FCMD brains than in the control brains (Fig. 5A, C).

### GAD expression is enhanced by *fukutin* knockdown in SH‐SY5Y cells

**Fig 6:**
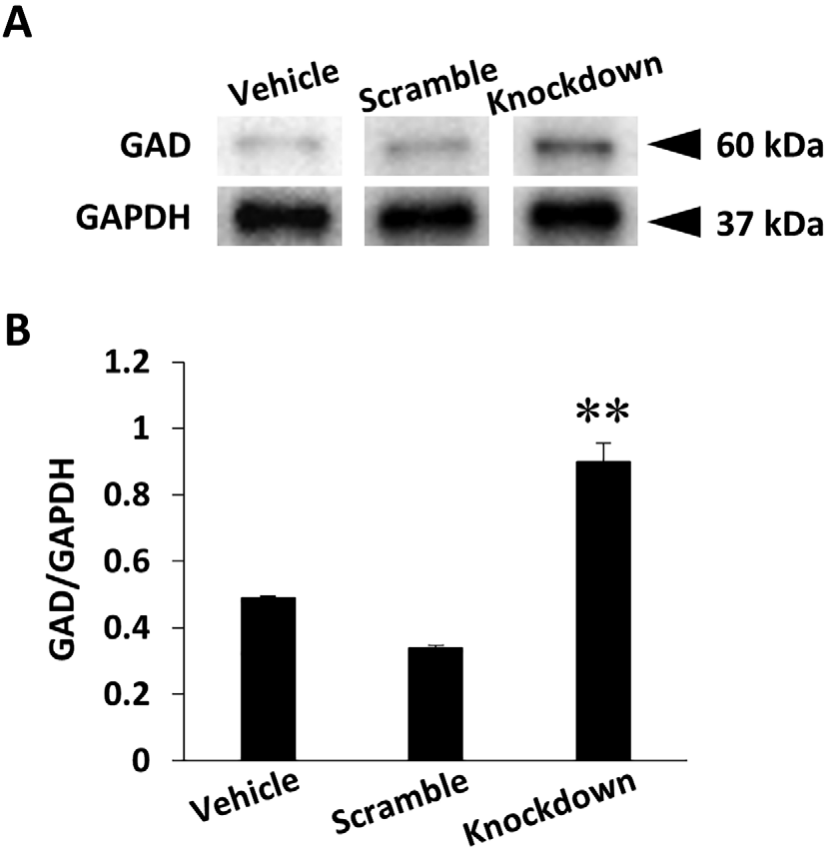
Results of Western blotting (A) and densitometry (B) for GAD in SH‐SY5Y cells with or without *fukutin* knockdown. (A) Immunoreactive signals for GAD in whole‐cell lysate are detected in each lane at a predicted mobility of 60 kDa on blots. (B) The GAD/GAPDH optical density ratio is significantly increased in the knockdown group as compared to the vehicle and scramble groups. *P* < 0.05 on one‐way ANOVA, **P < 0.01 *vs* the vehicle and scramble groups.

Based on the abovementioned morphological data in FCMD brains, we verified the influence of the *fukutin* expression status on GAD expression levels in SH‐SY5Y cells. As a consequence, GAD‐immunoreactive signal bands in whole cell lysate were identified in each lane at a predicted mobility of 60 kDa on Western blots (Fig. 6A). The GAD/GAPDH optical density ratio was significantly increased in the knockdown group as compared to the vehicle and scramble groups (Fig. 6B).

### Fukutin is colocalized with GAD and synaptophysin in human brains and SH‐SY5Y cells

**Fig 7:**
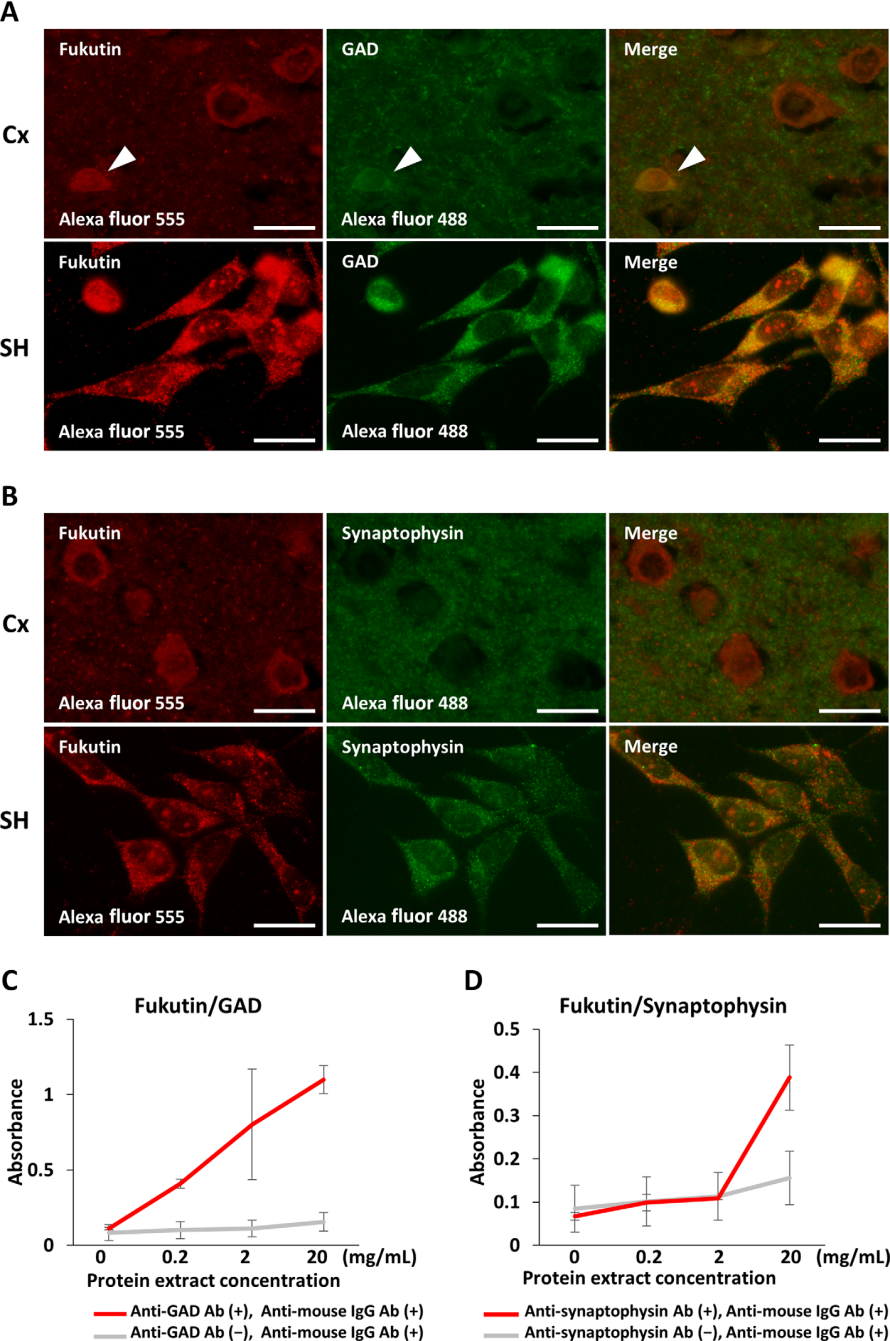
Representative findings of immunohistochemical and immunocytochemical localizations of fukutin, GAD, and synaptophysin in the cerebral cortex (Cx) from a 29‐year‐old control subject and cultured SH‐SY5Y (SH) cells (A, B) as well as evidence of binding affinity of fukutin to GAD or synaptophysin in SH cells obtained by a sandwich ELISA technique (C, D). (A, B) On double‐labeled immunofluorescence staining, immunoreactivities for fukutin (red) and GAD (green) are colocalized in the somatic cytoplasm (yellow) of neurons in the Cx and SH cells (A). Immunoreactivities for fukutin (red) and synaptophysin (green) are colocalized in the neuropil (yellow) of the Cx and in the cytoplasm of SH cells (B). (C, D) On sandwich ELISA, absorbances indicative of the formation of the fukutin/GAD complex (C) and the fukutin/synaptophysin complex (D) are significantly increased in a manner dependent on cell lysate concentrations of SH cells. *P* < 0.001 on one‐way ANOVA (C, D). Scale bars: 20 μm (A, B).

On double‐labeled immunofluorescence staining, we compared immunohistochemical and immunocytochemical localizations of fukutin and GAD or synaptophysin in the cerebral cortex and SH‐SY5Y cells. Both fukutin and GAD immunoreactivities were colocalized in the somatic cytoplasm of cortical neurons and SH‐SY5Y cells (Fig. 7A). In contrast, both fukutin and synaptophysin immunoreactivities were colocalized in the neuropil of the cerebral cortex and in the cytoplasm of SH‐SY5Y cells (Fig. 7B). No immunoreactive signals were visible on negative reaction controls (data not shown).

### Fukutin binds to GAD and synaptophysin in SH‐SY5Y cells

Given the colocalization data of fukutin and GAD or synaptophysin, we verified their binding affinity in SH‐SY5Y cells using a sandwich ELISA technique. Immunoreactive signals indicative of the formation of the fukutin/GAD complex (Fig. 7C) and the fukutin/synaptophysin complex (Fig. 7D) were significantly increased in a manner dependent on cell lysate concentrations. Both of these signals were canceled by omission of an antibody against GAD or synaptophysin.

## DISCUSSION

Previous studies demonstrated the appearance of NFTs, identified with antibodies against p‐tau, in the brains of elderly FCMD patients.^18^, ^19^ In the present study, NFTs, identified with an anti‐p‐tau antibody, were most frequently observed in the brain of a 27‐year‐old FCMD patient (case 3). In this case, polymicrogyria was seen in the brain, except in the occipital lobe; the occipital region displayed almost normal morphology. These observations suggest that the occurrence of polymicrogyria is closely relevant to the appearance of NFTs. To identify the type of neurons bearing NFTs, we conducted double‐labeled immunohistochemistry using antibodies against p‐tau and GAD; the latter is a marker of neurons that produce and release γ‐aminobutyric acid (GABA), as a inhibitory neurotransmitter, so‐called GABAergic neurons. As a consequence, p‐tau immunoreactivity was localized in the somatic cytoplasm of non‐GABAergic neurons, indicating that both of the proteins are exclusively distributed each other. Since most neurons in the cerebral cortex are excitatory,^27^, ^28^ it is likely that NFTs are formed in excitatory neurons. Furthermore, cortical neurons are irregularly arranged in the area of macroscopically observed polymicrogyria, which was proven by the Golgi's silver impregnation method.^29^ In the cerebrum of FCMD fetuses, a number of the subarachnoid neurons, which had overmigrated through the disrupted glia limitans, are immunoreactive for p‐tau.^19^ These observations suggest that there is a close link between the occurrence of polymicrogyria/cortical dysplasia and the appearance of NFTs in excitatory neurons in FCMD.

We next evaluated the influence of *fukutin* expression status on the tau phosphorylation status, using a cultured neuroblastoma cell line SH‐SY5Y. On immunofluorescence staining, tau phosphorylation was enhanced by *fukutin* knockdown of SH‐SY5Y cells and suppressed by *fukutin* overexpression of the neural‐differentiated cells. Consistently, Western blot analysis revealed a significant increase in p‐tau immunoreactivity in SH‐SY5Y cells processed with *fukutin* knockdown. However, we failed to detect quantitatively p‐tau in the neural‐differentiated cells with or without *fukutin* overexpression on blots probably due to a technical limitation for the cells. There are several protein kinases that catalyze the phosphorylation of tau in the human brain. The representative tau kinases are p25‐mediated cyclin‐dependent kinase 5 (Cdk5), mitogen‐activated protein kinase (MAPK), and GSK‐3β.^30^, ^31^, ^32^ Among them, GSK‐3β is one of the most active serine/threonine tau kinases^31^, ^33^, ^34^, ^35^ and is activated by phosphorylation at codon 216 tyrosine residue.^33^, ^34^, ^35^, ^36^ GSK‐3β is activated in association with aging, inflammation, mild cognitive impairment, and AD.^33^ Based on the background, we subsequently focused on GSK‐3β, a potential tau kinase in FCMD. Immunofluorescence staining revealed that GSK‐3β phosphorylation was enhanced by *fukutin* knockdown pf SH‐SY5Y cells and suppressed by *fukutin* overexpression of the neural‐differentiated cells. Consistently, Western blot analysis detected a significant increase in p‐GSK‐3β immunoreactivity in SH‐SY5Y cells processed with *fukutin* knockdown. However, we failed to detect quantitatively p‐GSK‐3β in the neural‐differentiated cells with or without *fukutin* overexpression on blots probably due to a technical limitation for the cells. These observations could indicate that fukutin drives tau phosphorylation that is catalyzed by phosphorylation‐activated GSK‐3β.

If the abovementioned phenomena actually occur, fukutin, GSK‐3β, and tau might be accumulated in the same subcellular fraction of a neuron. An earlier experimental study described that fukutin was localized in the Golgi apparatus in cultured myoblasts transfected with wild‐type *fukutin*.^4^ Subsequent *in vivo* studies using immunohistochemical approaches demonstrated that fukutin was localized in the cytoplasm and nucleus of neurons,^17^ as well as retinal cells.^37^ Other *in vitro* studies using immunocytochemistry and Western blotting showed that fukutin was localized in the endoplasmic reticulum (ER), cytoplasm, and nucleus of cultured cells.^37^, ^38^ Moreover, PSORT II, a website software, predicted cytoplasmic, mitochondrial, and nuclear localizations of fukutin in cultured cells. It is well‐known that both tau and GSK‐3β are ubiquitously expressed in several cell types and localized in the cytoplasm.^39^, ^40^ Thus, it is likely that fukutin interacts with GSK‐3β and tau in the cytoplasm of SH‐SY5Y cells.

Interestingly, we found a significant increase in the density of GAD‐immunoreactive neurons in the cerebral cortex of the FCMD brains. As an explanation for this finding, the increased density of GAD‐immunoreactive neurons may be an apparent impression attributed to a reduction in the neuropil volume of cortical lesions that include cell processes originating from non‐GAD‐immunoreactive neurons, which can bear NFTs and be destined to die. However, another finding of increased immunostaining intensity for GAD in the neuropil of elderly FCMD patients postulates the hypothesis that fukutin suppresses GAD expression. To test this hypothesis, we investigated the effects of *fukutin* knockdown on GAD expression status in SH‐SY5Y cells. Consequently, the *fukutin* knockdown‐driven enhancement of GAD expression in SH‐SY5Y cells on Western blots indicates that fukutin downregulates GAD expression. This is consistent with the finding of the increased GAD staining intensity in the neuropil of the FCMD brains. Such imbalance in the population of excitatory and inhibitory neurons is likely to be responsible for neurological disorders, including epilepsy;^27^ this is consistent with a clinical manifestation that more than 50% of FCMD cases develop epileptic episodes.^3^ However, there is no precedent showing similar results or convincing explanations for this issue.

Previously, the DGC was reported to be present in synaptic vesicles of GABAergic neurons but not excitatory neurons.^23^, ^25^ In addition, β‐DG, a component of the DGC, coexists with GAD in the inhibitory synapse,^23^ and fukutin has close relevance to α‐DG glycosylation.^5^ These observations are consistent with our *in vivo* and *in vitro* findings that fukutin is colocalized with GAD and synaptophysin, the latter being a specific marker for presynaptic vesicles^41^ in human neurons and SH‐SY5Y cells, suggesting that fukutin can be localized in presynaptic vesicles of GABAergic neurons. Moreover, our sandwich ELISA of SH‐SY5Y cells provided *in vitro* evidence of fukutin binding affinity to GAD and synaptophysin. This points to the possibility that fukutin plays a putative role in presynaptic function of GABAergic neurons through the formation of the fukutin/GAD complex and the fukutin/synaptophysin complex.

The results obtained from the present study are summarized as follows: (i) there is an inverse relationship between fukutin expression status and GSK‐3β/tau phosphorylation status; (ii) fukutin binds to GSK‐3β and tau; (iii) tau phosphorylation occurs in non‐GABAergic neurons; (iv) GAD expression is enhanced in the absence of fukutin; and (v) fukutin binds to GAD and synaptophysin in presynaptic vesicles of GABAergic neurons. These are novel properties of fukutin, being different from α‐DG glycosylation activity, and are closely associated with the appearance of NFTs in FCMD brains and the occurrence of epilepsy in FCMD patients. Finally, detailed mechanisms by which fukutin regulates the phosphorylation status of GSK‐3β and the expression status of GAD and also participates in presynaptic function of GABAergic neurons remain to be determined. Answers to these questions require further investigations.

## DISCLOSURE

The authors have no conflicts of interest to declare for the present study.
